# An *in vivo* high-throughput screening for riboswitch ligands using a reverse reporter gene system

**DOI:** 10.1038/s41598-017-07870-w

**Published:** 2017-08-10

**Authors:** Marion Kirchner, Kenji Schorpp, Kamyar Hadian, Sabine Schneider

**Affiliations:** 1 Center for Integrated Protein Science at the Department of Chemistry Technische Universität München, Lichtenbergstraße 4, 85748 Garching, Germany; 2 Assay Development and Screening Platform at the Institute for Molecular Toxicology and Pharmacology, Helmholtz Zentrum München für Gesundheit und Umwelt, Ingolstädter Landstraße 1, 85764 Neuherberg, Germany

## Abstract

Riboswitches are bacterial RNA elements that regulate gene expression in response to metabolite or ion abundance and are considered as potential drug targets. In recent years a number of methods to find non-natural riboswitch ligands have been described. Here we report a high-throughput *in vivo* screening system that allows identifying OFF-riboswitch modulators in a 384 well bioluminescence assay format. We use a reverse reporter gene setup in *Bacillus subtilis*, consisting of a primary screening assay, a secondary assay as well as counter assays to detect compounds in a library of 1,280 molecules that act on the guanine-responsive *xpt* riboswitch from *B. anthracis*. With this *in vivo* high-throughput approach we identified several hit compounds and could validate the impact of one of them on riboswitch-mediated gene regulation, albeit this might not be due to direct binding to the riboswitch. However, our data demonstrate the capability of our screening assay for bigger high-throughput screening campaigns. Furthermore, the screening system described here can not only be generally employed to detect non-natural ligands or compounds influencing riboswitches acting as genetic OFF switches, but it can also be used to investigate natural ligands of orphan OFF-riboswitches.

## Introduction

**Figure 1:**
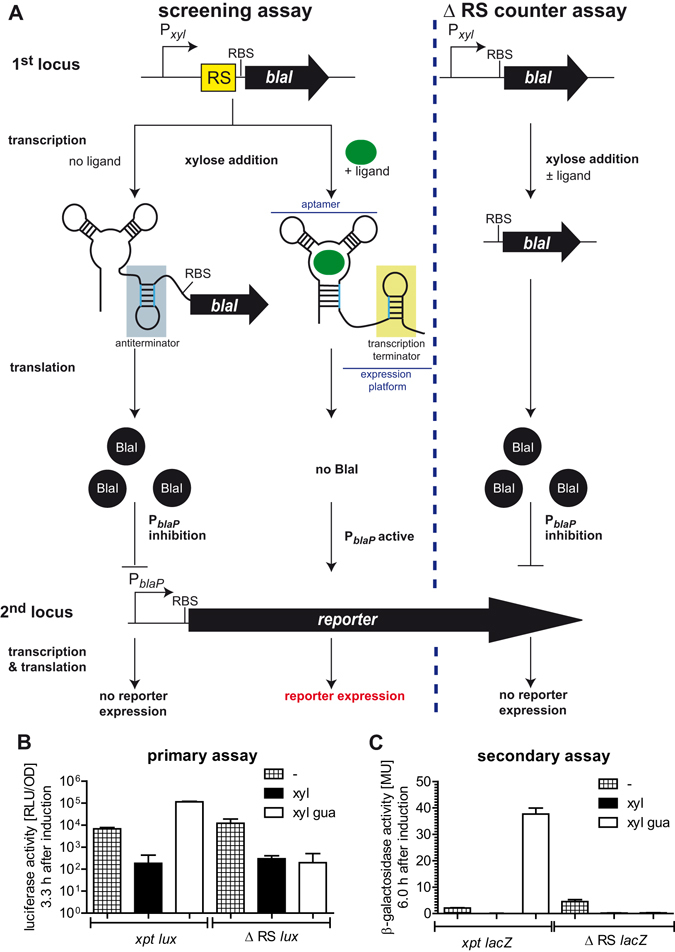
Schematic representation and functional characterization of the reporter gene-based screening for riboswitch activating compounds. (**A**) In the screening assay (left), the xylose-responsive promoter (P_*xyl*_; black tilted arrow) is located upstream of a transcriptional fusion of the sequence encoding the riboswitch (RS = riboswitch; yellow box) and the *blaI* repressor gene (black arrow; RBS = ribosome binding site). The riboswitch consists of a ligand-binding aptamer domain and an expression platform (blue) where the transcription terminator (light green) is located. Upon addition of xylose, transcription is induced and results in a riboswitch-*blaI* fusion mRNA. The repressor protein BlaI (black circles) inhibits the P_*blaP*_ promoter and hence the expression of reporter genes (black arrow). In the presence of a riboswitch ligand (green circle), ligand binding to the riboswitch aptamer leads to the formation of a transcriptional terminator. Thus, no BlaI proteins are produced, resulting in reporter gene expression. In the ∆ RS counter assay (right), *blaI* transcription cannot be blocked by the riboswitch ligand. (**B**) Verification of the primary screening assay using the guanine-dependent *xpt* riboswitch from *B. anthracis* and counter assay with the luciferase reporter genes. The luciferase activity [RLU/OD] (logarithmic scale) was obtained 3.3 h after induction. Addition of xylose leads to a reduction of luciferase activity (no xylose: checked bars; with 0.01% (w/v) xylose: black bars). The luciferase activity is restored through the addition of guanosine in the strains containing the riboswitch, but not in the control strain without riboswitch (Δ RS) (0.01% (w/v) xylose; 1 mM guanosine; white bars). (**C**) Verification of the secondary assay and its corresponding counter assay with β-galactosidase as reporter. β-galactosidase activities (Miller units (MU); linear scale) were determined 6 h after induction. Addition of guanosine to the growth media restores reporter gene expression specifically in the strain containing the *B. anthracis xpt* riboswitch. The assays were carried out in the presence of 0.01% (v/v) DMSO. The mean and standard deviations of three independent biological replicates are given. Please note, due to its higher solubility guanosine was used instead of guanine.

Structured RNA elements are important and quite unexplored drug targets. Since its discovery, RNA was thought to act merely as intermediate infrastructural component (ribosomal RNA, transfer RNA) and messenger (mRNA) between genes and proteins. However, in the last two decades, RNAs have proved to be tremendously versatile molecules. Due to their ability to acquire complex three-dimensional structures they fulfill functions almost as multifaceted as those of proteins and play a pivotal role in numerous cellular key processes (see ref. 1 for a review). One example are riboswitches, which are structured cis-acting regulatory RNA elements present in the 5′ untranslated region of mRNAs and are almost exclusively found in archaea or bacteria. Bacteria use them to link the bioavailability of metabolites, such as nucleobases and amino acids, as well as ions to the expression of genes encoding for their synthesis and transport^2–4^. In the gram-positive bacterium *Bacillus subtilis* (*B. subtilis*) riboswitches control the expression of about 2% of all genes and thereby regulate a variety of biochemical pathways^5^. Generally, riboswitches consist of two parts called aptamer domain and expression platform (Fig. 1A). The aptamer domain binds the respective riboswitch ligand with high selectivity, which induces a conformational change in the expression platform, leading to different gene-regulatory outcomes. Thus, depending on the riboswitch, the abundance of the ligand can induce or prevent transcription as well as translation, or regulate the RNA stability (see ref. 2 for a review).

Riboswitches have been acknowledged as potential drug targets^6, 7^ due to their ability to bind small molecules with high affinity and selectivity. Furthermore, riboswitches are almost unique to bacteria and archaea, where they often regulate the expression of proteins important for pathogenicity or survival. Several antibacterial compounds such as L-aminoethylcysteine, 2,5,6-triaminopyrimidin-4-one and roseoflavin are known to bind riboswitches although their mechanism of action is not always solely due to riboswitch binding^8–11^.

In the last decade, natural and synthetic ligand analogues have been identified by means of *in vitro* screening methods, structure-based computational docking as well as phenotypic screening targeting flavin mononucleotide (FMN), *glmS*, T-box or S-adenosylmethionine (SAM) riboswitches^12–16^. An indirect *in vivo* high-throughput screening for fluoride toxicity agonists using an ON fluoride riboswitch in fusion with *lacZ* as a reporter for the intracellular fluoride concentration was reported^17^. Another recently published *lacZ*-based *in vivo* screening method in *Escherichia coli* was aimed to identify thiamine pyrophosphate (TPP) riboswitch ligands in 96 well format^18^.

Compared to *in vitro* screening systems, *in vivo* screening setups have three major advantages: They not only select against molecules toxic for cells in general, but also for compounds that are able to enter cells. Furthermore, they display interactions in a physiologically relevant environment. In contrast to ON riboswitches, which enhance gene expression upon ligand binding, ligand binding to OFF riboswitches causes a reduced gene expression. When searching OFF riboswitch agonists, compounds interfering with signal generation will appear as false-positive hits^19^. Therefore, it is beneficial to reverse the reporter readout, to monitor a reduction in gene expression as signal increase.

To revert the reporter readout, we have previously established a reverse reporter gene system in the gram-positive model organism *B. subtilis* that enabled us to elucidate OFF riboswitch function^20^. The system consists of two parts which are integrated into two different loci in the *B. subtilis* W168 genome (Fig. 1A, left): The first part comprises the sequence encoding for the target riboswitch as well as the *blaI* gene encoding for the BlaI protein from *B. licheniformis*, which are both placed under control of the xylose-inducible promoter P_*xyl*_. Consequently, transcription is induced if the promoter P_*xyl*_ is activated with xylose. The second part is a luciferase reporter encoded by the *luxABCDE* genes controlled by the promoter P_*blaP*_. The crux of this system is the ability of the transcriptional repressor BlaI to downregulate activity of the P_*blaP*_ promoter. Thus, if the promoter P_*xyl*_ is activated by xylose addition, the nascent mRNA contains the riboswitch followed by *blaI*. If the transcriptional OFF-riboswitch binds its ligand, a conformational change leads to the formation of a transcription terminator at the end of the riboswitch. Due to the transcriptional stop no mRNA encoding *blaI* and no BlaI-proteins are produced. Therefore, in the presence of a riboswitch-activating compound and transcriptional inhibition, BlaI is absent. Without BlaI, the promoter P_*blaP*_ is active and a positive readout of the reporter gene expression can be detected. Advantages here are for one that the bioluminescence signal can be monitored without cell lysis in parallel to the bacterial growth over time. Additionally, a high signal/noise ratio can be expected due to a beneficial concentration ratio between parts integrated in the genomic DNA and RNA/proteins. With this reverse reporter gene system we have characterised the response and ligand specificity of five different guanine riboswitches from the pathogen *B. anthracis*, the causative agent of anthrax^20^. We could show that the *xpt* riboswitch from *B. anthracis* directly binds guanine and regulates reporter gene expression in response to guanine and hypoxanthine. Like its homologue from *B. subtilis* this riboswitch presumably regulates in its natural context the expression of a xanthine phosphoribosyl transferase (Xpt) and a xanthine permease (PbuX), which are involved in the uptake and metabolism of purines^21^. Previously it was argued that the guanine metabolism is important for the pathogenicity and survival of *B. anthracis* ^8, 11, 22^.

In the present study, we describe the adaption of this reporter gene system for high-throughput screening to identify activators of the guanine-responsive *B. anthracis xpt* riboswitch from large compound libraries. In order to carry out this luminescence-based screening assay in 384 well plates parameters such as for example induction time and signal window needed to be optimized, but also the reproducibility and robustness had to be analysed. Since compound libraries are commonly solubilized in dimethylsulfoxid (DMSO), we also tested the tolerance of our setup for DMSO. The system was then used to screen a compound library consisting of 1,280 U. S. Federal Drug Administration (FDA)-approved drugs^23, 24^. Here we could obtain four initial hits. For one of them, the nucleoside analogue gemcitabine, the increase in relative reporter gene expression could not only be reproduced, but also be confirmed in the orthologous secondary reverse reporter gene assay, which was based on β-galactosidase. Using counter assays we could additionally demonstrate that this effect is dependent on the presence of the *xpt* riboswitch, albeit this might be due to its influence on nucleoside metabolism rather than direct binding to the riboswitch.

In summary, we have established a reporter gene assay that allows us to identify compounds from large libraries that impact on riboswitch-mediated gene regulation. Using a small library of FDA-approved drugs, we could demonstrate the capability of our screening assay for bigger HTS campaigns. Furthermore, the system is not restricted to a particular riboswitch sequence and theoretically any riboswitch whose activation results in shut-down of gene expression can be incorporated and screened for ligands.

## Results and Discussion

To be able to identify novel ligands of a *B. anthracis* riboswitch, we established an *in vivo* screening in the model organism *B. subtilis* based on reporter gene expression. We chose the gram-positive model organism *B. subtilis* as chassis for our screening because it is a safe organism, which is phylogenetically related to *B. anthracis*. There are convenient protocols for genetic modifications, which we used to create four different *B. subtilis* strains containing two genetically engineered loci in their genome and differed in the presence of the riboswitch in one locus and in their reporter gene in the other locus.

The bioluminescence readout generated by the expression of the *luxABCDE* genes has several advantages: (i) It can directly be measured *in vivo* in time-course measurements without preceding cell lysis. (ii) It is self-regenerating and does not rely on external substrate addition^25^. (iii) It displays a very low background in *B. subtilis* and does not interfere with the determination of the bacterial growth rate at an optic density of 600 nm (OD_600_). (iv) The OD_600_ gives additional information about the fitness of the bacteria, a relevant parameter for compound screening. Thus, we decided to use the *luxABCDE* genes as reporters in the primary high-throughput screening. For the validation of the obtained hits, we constructed a secondary assay using β-galactosidase (*lacZ*) as different reporter gene. The two reporter assays clearly differ in the production of their output: while bioluminescence is produced during oxidation of myristyl aldehyde with FMNH_2_ consumption, β-galactosidase activity is determined through *ortho*-nitrophenyl-β-galactoside (ONPG) hydrolysis^26–28^. They can be regarded as orthogonal and hence are well-suited as primary and secondary assays.

**Figure 2:**
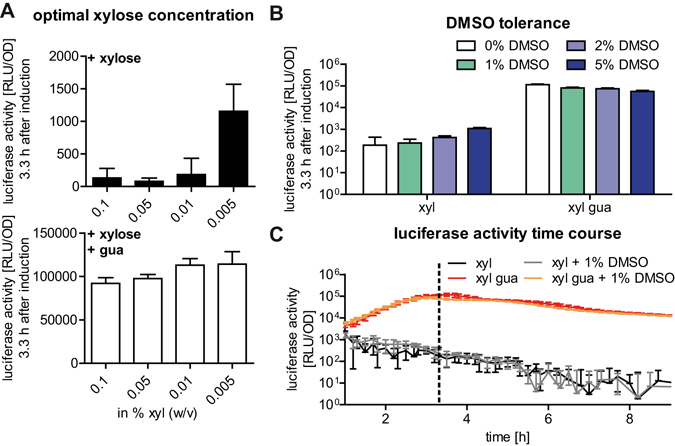
Optimal xylose concentration and DMSO tolerance. (**A**) Linear plots of the relative luciferase activities [RLU/OD] of the *xpt* RS *lux* reporter strain 3.3 h after redilution and induction with different concentrations of xylose (0.1%, 0.05%, 0.01%, 0.005% (w/v)), in the absence (top; black bars) and presence of 1 mM guanosine (bottom; white bars). (**B**) Influence of DMSO (0–5% (v/v)) on the luciferase activity [RLU/OD] of the *xpt* RS *lux* reporter strain in the presence of 0.01% (w/v) xylose (left), or 0.01% (w/v) xylose and 1 mM guanosine (right). Samples without DMSO are shown in white; samples with 1%, 2% or 5% DMSO in turquoise, light blue or blue. The relative luciferase activity is plotted on a logarithmic scale. (**C**) Time course of the relative luciferase activity [RLU/OD] of the *xpt* RS *lux* reporter strain in the presence of 0.01% (w/v) xylose (black curve), 0.01% (w/v) xylose and 1 mM guanosine (red), without as well as with 1% (v/v) DMSO (grey and orange, respectively). Luminescence and cell densities were measured 1–9 h after induction and the average RLU/OD values of three independent experiments with their standard deviations determined. The 3.3 h mark is indicated by the dashed line. The relative luciferase activity is plotted on a logarithmic scale.

To verify the system we used two *B. subtilis* strains both containing the P_*xyl*_-*B. antracis xpt* riboswitch-*blaI* part and either the P_*blaP*_-*luxABCDE* or the P_*blaP*_-*lacZ* reporter part, respectively (*xpt* RS *lux*; x*pt* RS *lacZ)*. For both reporter genes we found that xylose addition (xyl; black bars) leads to downregulation of the luciferase and β-galactosidase activity to a basal level (Fig. 1B,C). Addition of xylose plus guanosine (xyl gua; white bars) enhances reporter gene expression more than 500-fold (luciferase) and ~1000-fold (β-galactosidase), respectively. The corresponding signal windows were 8,6 (β-galactosidase) and 13.4 (bioluminescence). Due to the higher solubility of guanosine compared to guanine, guanosine was used instead of the riboswitch ligand guanine. Guanosine is transported into the cells and converted to guanine intracellularly^29–31^. The maximal luciferase activity upon guanosine induction was observed at the onset of the stationary phase (3.3 h after induction; Fig. 2C) and the β-galactosidase activity gave a strong signal 6 h after induction.

To be able to verify if changes in reporter gene expression are indeed caused by the activity of the riboswitch, strains containing the reporter system excluding the riboswitch sequence (Δ RS *lux*; Δ RS *lacZ*) were generated (=counter assay, Fig. 1A right). Indeed here the addition of guanosine does not alter or even restore the expression of either reporter gene (Fig. 1B,C). These findings demonstrate that the reporter gene setup works as designed. Possible false-positive hits could arise from off-target effects caused, for instance, by cross-reactive compounds binding to the *B. anthracis xpt* riboswitch and to *B. subtilis* purine riboswitches, thus generally affecting the overall bacterial growth. In addition, compounds that might act on the *xpt* riboswitch, but are overall toxic to cells could be missed. In order to minimize toxic effects and ensure optimal growth of our reporter strains, we chose a medium that allowed *B. subtilis* cells to grow in the presence of previously reported antibacterial guanine analogues^8, 32^.

To identify novel ligands for the *B. anthracis xpt* riboswitch from large compound libraries, our reverse reporter gene system established previously in *B. subtilis* ^20^ (Fig. 1A) needed to be adapted for high-throughput screening. First we optimized the observed signal-to-noise (S/N) ratio for the luciferase reporter by testing xylose concentrations ranging from 0.005–0.01% (w/v) in the absence and presence of guanosine (Fig. 2A). Sole addition of xylose resulted in a comparable reduction of relative luciferase activity for 0.01–0.1% (w/v) xylose concentration. The lowest xylose concentration (0.005% (w/v)) displayed a considerably higher residual reporter gene activity. Even in the presence of the highest xylose concentration (0.1% (w/v)) addition of guanosine restored reporter gene expression, proving the stability of the system. We chose to further use a xylose concentration of 0.01% (w/v) since it was the lowest xylose concentration yielding a good signal-to-noise (S/N) ratio of about 500.

Small-molecule screening libraries typically consist of compounds dissolved in DMSO. As it is known that DMSO can not only affect cellular growth^33^, but also RNA structures and RNA-ligand interactions^34, 35^ it had to be ensured that the reporter gene assay tolerates the presence of at least 1% (v/v) DMSO. Thus, the luciferase activity upon induction with 0.01% (w/v) xylose and guanosine was determined in the presence of 1–5% (v/v) DMSO (Fig. 2B). The results demonstrated that 1% and 2% (v/v) DMSO reduced the signal-to-background (S/B) ratio by 40% and 70%, respectively. Nevertheless, the corresponding S/N ratios were greater than 750 and Z-factors > 0.75. Consequently the screening system can tolerate up to 2% (v/v) DMSO without a strong negative impact on the readout. The curve progressions in the presence or absence of 1% (v/v) DMSO were almost identical (Fig. 2C).

**Figure 3:**
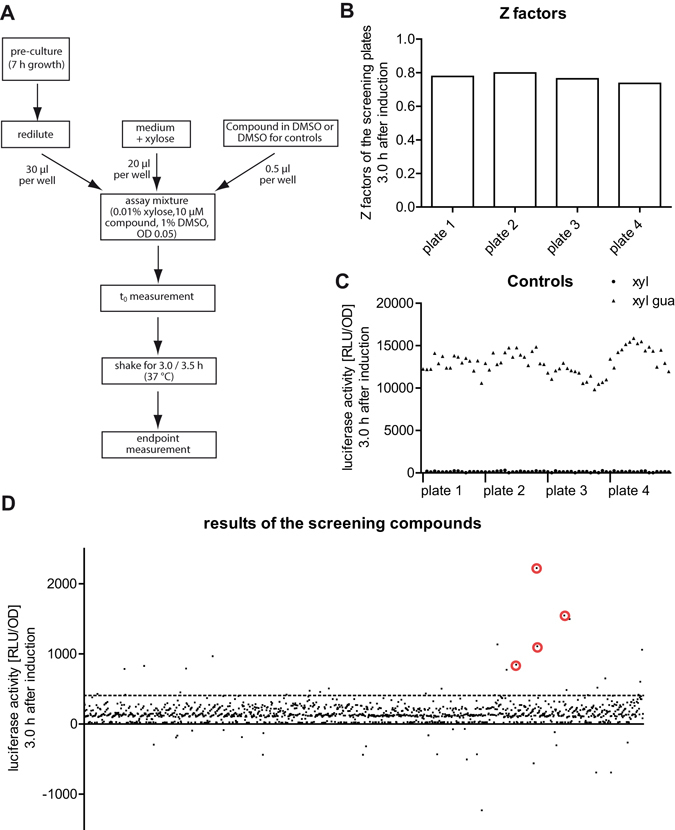
High-throughput screening assay. (**A**) Scheme of the high-throughput assay procedure. Cells from a pre-culture were diluted in medium supplemented with xylose to a final OD_600_ of about 0.05. After addition of the compounds, the starting (t_0_) OD_600_ and luminescence were measured for each well. After incubation at 3.0 and 3.5 hours at 37 °C with agitation, the final OD_600_ and luminescence were measured. (**B**) Z factors and (**C**) relative luciferase activity [RLU/OD] of wells containing positive (triangles) and negative controls (dots), calculated 3 h after induction. (**D**) Plot of the relative luciferase activity [RLU/OD] of all compounds in the screening. The compounds selected as hits are marked by red circles. The threshold for selection criterion 1 (mean RLU/OD + 4 × standard deviation of the minimum control) is indicated by a dashed line.

To adapt the system further to high-throughput assay conditions, the setup was changed from 96 well to 384 well plates, which were analysed in end-time measurements 3 and 3.5 h after induction. First, the reproducibility was tested in 384 well plates using the maximum (xyl gua) and minimum (xyl) controls. Three independent experiments yielded Z factors of 0.72 ± 0.09, S/B ratios of 321 ± 155 and S/N ratios of 210 ± 57, with a signal window of 10 +/− 5, demonstrating that the assay quality fulfils screening criteria and gives reproducible results. Therefore, this setup was used for screening of a compound library consisting of 1,280 FDA-approved drugs. The final protocol for the screening (Fig. 3A) included two end-point measurements. In the screening data (Fig. 3C), the very good Z factors (0.77 ± 0.02; see Fig. 3B) and signal windows (SW = 10.87 ± 1.46) again confirmed the robustness of the system. The luciferase activities of the *xpt* RS *lux* reporter strain treated with the compounds are visualized in Fig. 3D.

With the screening data in hands it is important to establish an appropriate selection process to exclude false-positive and false-negative hits. False-positive hits can occur due to auto-luminescent compounds and compounds which inhibit *B. subtilis* growth and thereby cause high luciferase activity [RLU/OD] without elevated luminescence signals. Thus only compounds meeting the following selection criteria were considered as hits: First, the luciferase activity (luminescence/OD_600_ [RLU/OD]) had to exceed a threshold (mean RLU/OD + 4 × the standard deviation of the minimum control (σ_min_)) 3 h after beginning of the incubation. Second, the raw luminescence signal had to be higher than the mean RLU + 3 × σ_min_ to exclude compounds with background luciferase activity and a very low OD_600_. Third, only wells displaying an elevated luciferase activity (>(mean + 3 × σ_min_)) also 3.5 h after beginning of the incubation were considered, ensuring that the compounds cause a long-term signal. Fourth, compounds with high auto luminescence in the initial (t_0_) measurement (>(mean + 3 × σ_min_)) were excluded. Fifth, compounds in plate positions where the luminescence was generally higher due to the edge effect were carefully compared to the neighboring luminescence values and only wells with elevated luminescence were accepted as hits. Applying these criteria, the screening yielded four primary hits which are encircled in red in Fig. 3D. Common hit rates of less than 0.5% are observed in high-throughput screenings for agonist ligands targeting enzymes^19^. With 0.3% our final hit rate was satisfactory taking into account that guanine riboswitches are known to bind their ligands with high specificity^5^, and ligand binding needs to induce and stabilize a RNA conformation that impacts on transcription.

**Figure 4:**
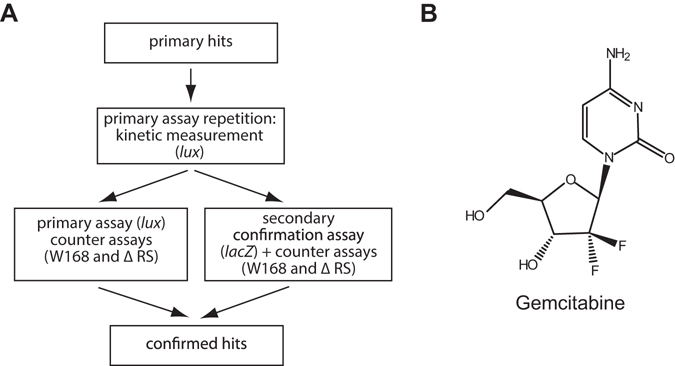
Scheme of primary hit verification and structure of gemcitabine. (**A**) For hit verification, primary hits were repeated using the primary assay and further analysed with the secondary and counter assays. (**B**) Structure of the hit compound gemcitabine (2′2′difluorodeoxycitidine).

**Figure 5:**
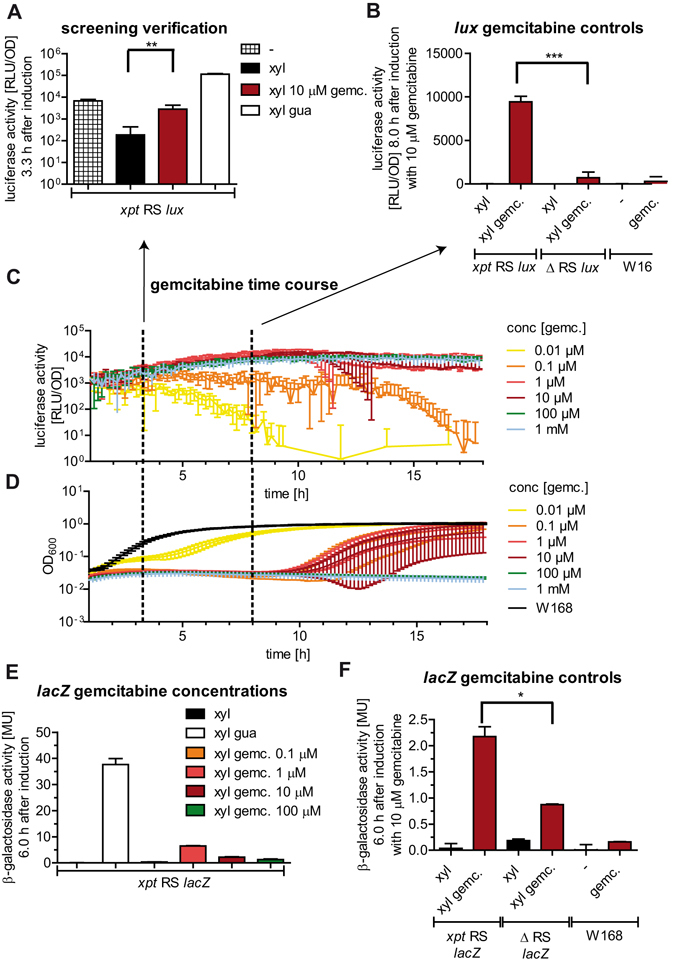
Verifications of the hit compound gemcitabine. (**A**) Luciferase activity [RLU/OD] (logarithmic scale) of *xpt* RS *lux* reporter strain 3.3 h after induction without treatment (plaid), treated with xylose (black), with xylose and gemcitabine (red), or xylose and guanosine (white). (**B**) Luciferase activity [RLU/OD] (linear scale) of the *xpt* RS *lux* reporter strain (left), the ∆ RS *lux* control strain (middle) and the wild type (W168; right) 8.0 h after induction. The *xpt* RS *lux* strain and the ∆ RS *lux* strain were either treated with xylose (black) or xylose and 10 µM gemcitabine (red). W168 was untreated (black) or treated with 10 µM gemcitabine (red). (**C**) Relative luciferase activity [RLU/OD] (logarithmic scale) of the *xpt* RS *lux* reporter strain treated with xylose and gemcitabine (0.01 µM (yellow), 0.1 µM (orange), 1 µM (red), 10 µM (dark red), 100 µM (green) and 1 mM (blue)) 1–18 h after induction. The time points 3.3 and 8 h are indicated by dashed lines. (**D**) Growth of the *xpt* RS *lux* reporter strain treated with xylose and 0.01 µM–1 mM gemcitabine, 1–18 h after induction. Colour code as in (**C**). The growth (OD_600_, logarithmic scale) of the wild type W168 is shown in black. (**E**) β-galactosidase activity (Miller units [MU]; linear scale) 6.0 h after induction of *xpt* RS *lacZ* with xylose (black), xylose and guanosine (white) or xylose and gemcitabine (0.1 µM (orange), 1 µM (red), 10 µM (dark red) or 100 µM (green)). (**F**) β-galactosidase [MU] (linear scale) of the *xpt* RS *lux* reporter strain (left), the ∆ RS *lux* control strain (middle) and the wild type (W168; right) 6.0 h after induction. The *xpt* RS *lux* strain and the ∆ RS *lux* strain were either treated with xylose (black) or xylose and 10 µM gemcitabine (red). W168 was untreated (black) or treated with 10 µM gemcitabine (red). If not mentioned otherwise, 1 mM guanosine and 0.01% (w/v) xylose were used. The average values of three independent experiments, with the respective standard deviations are shown.

Albeit for a number of compounds the relative luminescence activity was above the threshold of selection criterion 1(Fig. 3D), they did not meet the other four selection criteria. Thus the remaining four primary hits were validated according to the strategy depicted in Fig. 4A. First, the primary assay was repeated in a time-resolved manner, using three different compound concentrations (5, 10 and 20 µM) and the bioluminescence and the OD_600_ were recorded every ten minutes (data not shown). Here, only for one of the compounds, gemcitabine (Fig. 4B), the results of the HTS could be reproduced. During the screening, the well supplemented with gemcitabine showed an 8-fold higher relative luciferase activity compared to the negative control. In addition to causing bioluminescence, it did impair cell growth. In the repeated primary assay 10 µM gemcitabine caused a significantly higher signal 3.3 h after induction compared to the negative control xyl (P = 0.0077; Fig. 5A). To investigate the dose-dependent effect of gemcitabine on the luciferase activity we treated the *xpt* RS *lux* reporter strain with 0.01% (w/v) xylose and different concentrations of gemcitabine, ranging from 0.01 µM–1 mM (Fig. 5C,D). Compared to the wild type W168, all tested gemcitabine concentrations lead to growth defects with some dose-dependency in *B. subtilis* (Fig. 5D). For example, the treatment with 0.01 µM gemcitabine caused a growth delay of about 3 h. In the presence of 100 µM and 1 mM gemcitabine, growth of *B. subtilis* was completely inhibited. The relative luciferase activity (Fig. 5C) generally increased slowly after induction without displaying a real dose-dependency.

In order to test if the gemcitabine-mediated increase of relative luminescence is specific to the presence of the riboswitch we treated the control reporter strain lacking a riboswitch (Δ RS *lux*) and the wild type W168 with 10 µM gemcitabine and determined the relative luciferase activity. The results demonstrate that the presence of gemcitabine in the media overall increases the luminescence signal, even in the wild type without the luciferase reporter genes (Fig. 5B). Nevertheless, in the presence of gemcitabine the relative luminescence of the *xpt* RS *lux* strain is significantly higher than that of the Δ RS *lux* sample (P < 0.0001) (Fig. 5B), proving that gemcitabine has an effect on the *xpt*-riboswitch- mediated gene regulation.

Furthermore, we used the secondary β-galactosidase (*lacZ*) reporter gene assay to verify the impact of gemcitabine in the primary assay. Treating the *xpt* RS *lacZ* reporter strain with 1, 10 and 100 µM gemcitabine resulted in an elevated β-galactosidase activity (Fig. 5E); however, the effect is not dose-dependent, as already seen with the luciferase reporter (Fig. 5C). Possibly, this could be due to the increase in gemcitabine toxicity at higher concentrations. We also compared the effect of gemcitabine on the *xpt* RS *lacZ* strain with its effect on the wild type and the ∆ RS *lacZ* strains (Fig. 5F). As for the luciferase reporter, an increase in β-galactosidase activity can be observed in the wild type and the ∆ RS *lacZ* reporter strain. But again, the reporter gene activity in the bacteria with the *xpt* riboswitch was significantly increased compared to the ∆ RS *lacZ* reporter strain (P = 0.0201) in the presence of gemcitabine (Fig. 5F). Thus, we could confirm the effect of gemcitabine on the riboswitch-regulated reporter gene expression with an orthogonal reporter gene assay. Gemcitabine is known to interfere with the *de novo* synthesis of nucleoside triphosphates through inhibition of the ribonucleotide reductase by its di-phosphorylated metabolite^36^. Therefore further work will be necessary to dissect the molecular mechanism of gemcitabine on the *xpt* riboswitch- mediated gene regulation.

In summary, we present here a reporter gene based high-throughput *in vivo* screening system in *B. subtilis* to identify synthetic riboswitch ligands based on bioluminescence. The assay setup demonstrated good Z factors and signal windows. Furthermore, the wide signal window and the simultaneous bioluminescence and cell-density measurements allowed us to choose stringent hit selection criteria. Gemcitabine, one of four primary hits identified, showed a reproducible *xpt* riboswitch-dependent increase in reporter gene expression in the primary as well as in an orthogonal secondary reporter gene assay. Nevertheless, the molecular mechanism of gemcitabine with regard to the *B. anthracis xpt* riboswitch, as well as any direct or indirect impact still needs to be investigated. However, this reverse reporter gene system could be generally applied to discover natural or synthetic ligands of known or orphan riboswitches, which turn off transcription or translation upon ligand binding.

## Material and Methods

Chemicals, enzymes and buffers were bought from Carl Roth (Karlsruhe, Germany), VWR (Radnor, USA), AMRESCO (Solon, USA), New England Biolabs (Ipswich, USA), Thermo Fisher (Karlsruhe, Germany) or Promega (Madison, USA). Compounds were purchased from TCI (Tokyo, Japan), ChemDiv (San Diego, USA), Sigma-Aldrich (St. Louis, USA), Molekula (Newcastle Upon Tyne, UK) or Thermo Fisher (Karlsruhe, Germany).

### Cloning procedures

For cloning purposes, bacteria were grown in Luria Bertani (LB) medium (0.5% (w/v) NaCl, 1% (w/v) peptone, 0.5% (w/v) yeast extract) at 37 °C with agitation. Enzymes were used according to manufacturer’s instructions. For scarless insertion of riboswitch parts into a plasmid golden gate cloning was used based on Engler *et al*., 2008^37^ and Engler *et al*., 2009^38^. Plasmid and inserts were amplified with primers containing a *Bsa*I recognition site and the desired restriction site using Phusion polymerase. The purified DNA fragments were incubated with *Bsa*I and a highly concentrated ligase (Roche) in CutSmart buffer for 30 cycles of 37 °C, 5 min. followed by 20 °C, 2 min before electro-transformation of *Escherichia coli*. The *E. coli* strains DH5α or XL1 blue were electro-transformed and selected using 100 µg/mL ampicillin.

The *B. subtilis* strains are based on W168 and were grown in the presence of 100 µg/mL spectinomycin and 5 µg/mL chloramphenicol when appropriate. *B. subtilis* transformations were performed as described by Radeck *et al*., 2013^39^: Briefly, MNGE-medium (52.1 mM K_2_HPO_4_, 38.5 mM KH_2_PO_4_, 2.85 mM MgSO_4_, 2.80 mM sodium citrate, 0.105 M glucose, 10.3 mM potassium glutamate, 39.9 μM ammonium ferric citrate, 233 μM tryptophan, 399 μM threonine) was inoculated to an optical density at 600 nm (OD_600_) of 0.1 using overnight cultures and grown at 37 °C under agitation until OD_600_ = 0.8–1.3. *B. subtilis* genomic DNA or *Sca*I-linearized plasmids were added to the competent cells which further grown for 1 h at 37 °C before yeast extract (final concentration 0.5% (w/v)), casamino acids (CAA; 0.5% (w/v)), tryptophan (0.23 mM) and, if required, chloramphenicol (77 nM), were added. After incubation for one hour at 37 °C, cells were plated on LB plates (LB medium plus 2% agar) with antibiotics. Integrations into the *thrC* locus or the *amyE* locus were verified using threonine auxotrophy tests in minimal medium or iodine starch tests.

### Luciferase assays

Day cultures were inoculated 1:100 in modified CSE medium from overnight cultures in LB medium with selection, if necessary, and incubated at 37 °C and 200 rpm until OD_600_ = 2–3 was reached. The cultures were rediluted to OD_600_ = 0.05 and guanosine (final concentration 1 mM), DMSO (1%, 2% or 5% (v/v), xylose (0.1–0.005% (w/v)) and gemcitabine (0.01 µM–1 mM) were added, as appropriate. 96 well plates (black, µ-clear, Greiner^R^ Bio-One, Frickenhausen, Germany) were filled with 100 µl per well and incubated at 37 °C with double orbital shaking (108 rpm) in a Spark^TM^ 10 M multiwell reader (Tecan, Grödig, Austria). The luminescence and absorbance at 600 nm were measured every ten minutes. The obtained bioluminescence and OD_600_ values were corrected using the values of medium only wells averaged over time. The relative luminescence units divided by the OD_600_ yielded the luciferase activity [RLU/OD].

### Dimethylsulfoxide tolerance

To test the impact on the presence of dimethylsulfoxide (DMSO) on the luciferase signal, 1–5% (v/v) DMSO was added and the bioluminescence determined, upon induction with 0.01% (w/v) xylose and guanosine (Fig. 2B). Here, a bioluminescence increase was observed in the xylose-treated samples with increasing DMSO concentrations. In contrast, the luciferase activity slightly decreased in the presence of xylose and guanosine with increasing DMSO concentrations. Addition of 5% (v/v) DMSO resulted in a low S/B ratio and some observable growth defects and thus was considered to be not suitable for the screening assay.

### High-throughput screening

For screening development, the screening itself and hit verification experiments a modified CSE medium based on MOPS buffer was used (40.0 mM MOPS, 25.0 mM (NH_4_)_2_SO_4_, 0.385 mM KH_2_PO_4_, 0.615 mM K_2_HPO_4_, 10.4 µM MnSO_4_, 0.50 mM MgSO_4_, 24.5 mM tryptophan, 42.0 mM threonine, 43.2 µM potassium glutamate, 84.0 µM ammonium ferric citrate, 37.0 µM sodium succinate, 139 µM fructose, 1% (w/v) casamino acids)^39^. For the high-throughput screening, overnight cultures were grown for about 7 hours at 37 °C with agitation in modified CSE medium plus antibiotic selection. 384 well plates (PS, black, µ-clear Greiner^R^ Bio-One, Frickenhausen, Germany) were filled with 50 µl per well using a MultiFlo^TM^ dispenser (BioTek, Winooski, USA). 16 wells per plate contained the following controls: cell suspension supplemented with 0.01% (w/v) xylose (=minimum control), 0.01% (w/v) xylose and 1 mM guanosine (=maximum control) modified CSE medium or modified CSE medium supplemented with 1 mM guanosine and 1% DMSO (v/v), respectively. All other wells were filled with modified CSE medium containing cells (OD_600_ = 0.05), xylose (0.01% (w/v)) and compounds solved in DMSO (final concentration 10 µM compound, 1% (v/v) DMSO). The compounds and DMSO were added using a Sciclone G3 liquid handling workstation (PerkinElmer, Waltham, USA). The initial luminescence and cell density (OD_600_) were determined with an EnVision multilabel reader (PerkinElmer, Waltham, USA) prior to incubation of the plates at 37 °C with agitation. Luminescence and OD_600_ values were finally measured after 3 and 3.5 h. For evaluation, the measured OD_600_ and luminescence values for the compound-containing wells were corrected by the averaged blank values (CSE medium-only containing wells). Finally, the adjusted luminescence values were divided by the OD_600_ of the same well (=luciferase activity [RLU/OD]).

### β-Galactosidase assays

For β-galactosidase assays, 10 ml modified CSE-medium supplemented with 0.01% xylose, 1 mM guanosine, 0.1% (v/v) DMSO, 0.1, 1, 10 or 100 µM gemcitabine and antibiotics, as appropriate, were inoculated to OD_600_ = 0.25 from overnight cultures and grown at 37 °C with agitation for 6 h before cell harvesting. Pellets were stored at −20 °C before usage. The assays were performed using a modified protocol based on Miller, 1972^28^: Pellets were resuspended in 60 mM Na_2_HPO_4_, 40 mM NaH_2_PO_4_, 10 mM KCl, 1 mM MgSO_4_, 20 mM β-mercaptoethanol, pH 7.0 and diluted to OD_600_ = 0.2–0.8. After OD_600_ measurement, lysozyme (0.12 mg/ml final concentration) was added and the solutions were incubated at 37 °C until they were clear. *Ortho*-Nitrophenyl-β-galactoside (ONPG) was added to a final concentration of 2 mM and the samples were incubated at room temperature. The reaction was stopped with 1 mM Na_2_CO_3_ after the samples turned yellow or 1 h after ONPG addition. The absorptions at 420 and 550 nm were measured and the Miller units (MU) were calculated according to the following formula:

$$MU=\,\frac{1000\ast (Ab{s}_{420}-(1.75\,\ast Ab{s}_{550}))}{(t\ast \,0.8\,ml\ast O{D}_{600})}$$

where Abs denotes the absorption and t the time from ONPG to Na_2_CO_3_ addition.

### Statistical analysis

Samples containing cells and 0.01% (w/v) xylose were used as minimum control and samples containing cells, 0.01% (w/v) xylose and 1 mM guanosine were used as maximum control. Average signal-to-noise (S/N) ratios and signal-to-background (S/B) ratios were calculated according to the formulas

$$\frac{S}{N}=\,\frac{mea{n}_{max}-\,mea{n}_{min}\,}{{\sigma }_{min}}\,{\rm{and}}\,\frac{S}{B}=\frac{mea{n}_{max}}{mea{n}_{min}}$$

where σ = standard deviation, max = maximal control and min = minimum control. Signal windows (SW) were determined according to the formula

$$SW=\,\frac{(mea{n}_{max}-\,mea{n}_{min})-3\ast ({\sigma }_{max}+{\sigma }_{min})\,}{{\sigma }_{max}}.$$

Z factors were calculated according to the formula

$$Z=\,1-\frac{3({\sigma }_{max}+{\sigma }_{min})}{|mea{n}_{max}-mea{n}_{min}|}.$$

P values were calculated with Prism (GraphPad, La Jolla, USA) using two-tailed student’s t-tests with Welch’s correction^40^.

### Data availability statement

The datasets generated during the current study are available from the corresponding author on reasonable request.

## Electronic supplementary material

Supplementary Information
