# Supplementary material for: An in vivo high-throughput screening for riboswitch ligands using a reverse reporter gene system
Source: Sci Rep. 2017 Aug 10;7:7732. doi: 10.1038/s41598-017-07870-w (PMC5552694; doi:10.1038/s41598-017-07870-w)
Supplement: Supplementary file 1 — Supplementary Information [file 41598_2017_7870_MOESM1_ESM.pdf]

An *in vivo* high-throughput screening for riboswitch ligands using a reverse reporter gene system

Marion Kirchner[1*], Kenji Schorpp[2], Kamyar Hadian[2] and Sabine Schneider[1*]

Supplementary table 1. Criteria: (1) mean RLU/OD > 4 x $\sigma_{min\ control}$ (t=3 h); (2) raw RLU > mean RLU + 3 x $\sigma_{min\ control}$; (3) mean RLU/OD > 4 x $\sigma_{min\ control}$ (t=3.5 h); (4) low auto luminescence at t_0; (5) no edge effect. "+" means the measurements meet the criterion. "-" means that the compound was excluded due to this criterion.

Compound	Criteria 1 (>407 RLU/OD)	Criteria 2	Criteria 3	Criteria 4	Criteria 5
**1**	429.38	-			
**2**	783.51	-			
**3**	417.18	-			
**4**	828.40	-			
**5**	449.70	-			
**6**	790.96	-			
**7**	965.52	-			
**8**	418.54	-			
**9**	473.87	-			
**10**	456.46	-			
**11**	504.98	-			
**12**	434.24	-			
**13**	503.14	-			
**14**	410.26	-			
**15**	1133.86	-			
**16**	771.80	-			
**17**	**845.28**	+	+	+	+
**18**	**2223.17**	+	+	+	+
**19**	**1107.94**	+	+	+	+
**20**	**1546.12**	+	+	+	+
**21**	483.38	-			
**22**	1495.33	-			
**23**	413.03	+	-		
**24**	517.60	+	-		
**25**	650.11	-			
**26**	416.22	+	-		
**27**	602.69	+	+	+	-
**28**	1058.27	+	+	-	

Supplementary table 2. *E. coli* and *B. subtilis* strains used in this study.

XL1 blue	*endA1 gyrA96*(*nal*R) *thi*-1 *recA1 relA1 lac gln*V44 F′[Tn10 *proAB*+ *lacI*q Δ(*lacZ*)*M15*] *hsdR17*(rK- mK+) *tet*R	Stratagene
DH5α	F– Φ80*lac*ZΔM15 Δ(*lac*ZYA-*arg*F) U169 *rec*A1 *end*A1 *hsd*R17 (rK–, mK+) *pho*A *sup*E44 λ– *thi*-1 *gyr*A96 *rel*A1	laboratory stock
W168	wild type, *trpC2*	laboratory stock
BS2	W168 *amyE*::pSB$_{BS}$1C-P$_{blaP}$-*lacZ*	this paper
BS41	W168 *amyE*::pSB$_{BS}$1C-P$_{blaP}$-*lux*	[17]
BS44 (*xpt* RS *lacZ*)	W168 *amyE*::pSB$_{BS}$1C-P$_{blaP}$-*lacZ thrC*::pXT-*B.ant_xpt*RS-*blaI*	this paper
BS47 (*xpt* RS *lux*)	W168 *thrC*::pXT-*B.ant_xpt*RS-*blaI amyE*::pSB$_{BS}$1C-P$_{blaP}$-*lux*	this paper
BS118 (Δ RS *lux*)	W168 *amyE*::pSB$_{BS}$1C-P$_{blaP}$-*lux thrC*::pXT-P$_{xyl}$-SD$_{B.ant}$ *xpt*RS-*blaI*	this paper
BS140 (Δ RS *lacZ*)	W168 *amyE*::pSB$_{BS}$1C-P$_{blaP}$-*lacZ thrC*::pXT-P$_{xyl}$-SD$_{B.ant}$*xpt*RS-*blaI*	this paper

Supplementary Table 3: Plasmids used in this study.

Name	Description	Construction / Reference
#157	pSB$_{BS}$1C-P$_{blaP}$-*lacZ*	[17]
#197	pSB$_{BS}$1C-P$_{blaP}$-*gfp*	[17]
#209	pSB$_{BS}$1C-P$_{blaP}$-*luxABCDE*	[17]
#171	pXT-BS-RS-*blaI*	[17]
#207	W168 *thrC*::pXT-*B.ant_xpt*RS-*blaI*	plasmid #171 amplified with primer pair o301/o302 and o259 cloned with golden gate cloning (*Bsa*I)
#253	*thrC*::pXT-P$_{xyl}$-SD$_{B.ant}$ *xpt*RS-*blaI*	pXT backbone amplified with o301/o363, cloned with golden gate cloning (*Bsa*I)

Supplementary Table 4. Primers and oligos used in this study. Restriction enzyme recognition sites are in italics, restriction sites are underlined.

o259	BaXptRS	TAT*GGTCTCA*ATCCAATAAATAGTTAGCTACACTCATATAATCGCGGGGATATGGCCTGCAAGTTTCTACCGAAGTACCGTAAATACTTTGACTATGAGTGAGGACGAATATATTTGCTTGTTTAGCATTCTTTTTTGCGAAACTCCAAAAGCGCGTCTCTCACTTGTAACGAGTGGTGGCGGCTTTTGGAGTTTTTTTATTGCATAAGAGGGGGAACAAACATGAA*GAGACC*ATT
o301	pXT-*blaI*GGCFwd	TAT*GGTCTCA*ATGAAAAAAATACCTCAAATCTCTGATGC
o302	pXT-P$_{xyl}$GGCRev	TAT*GGTCTCA*GGATCCTCTAGAGTCGACCTGC
o363	dRSSD$_{xptB.ant_}$ctr_rev	TAT*GGTCTCA*TCATGTTTGTTCCCCCTCTTGGATCCTCTAGAGTCGAC
